# Supplementary material for: Near-Infrared Spectroscopy as a Tool for the Traceability Control of High-Quality Iberian Dry-Cured Meat Products
Source: Foods. 2025 Jan 28;14(3):432. doi: 10.3390/foods14030432 (PMC11817402; doi:10.3390/foods14030432)
Supplement: Supplementary file 1 [file foods-14-00432-s001.zip › foods-3391887-supplementary.pdf]

Supplementary materials

Table S1. PLS-DA calibration results of models developed by means of MicroNIR™ 1700 OnSite-W (VIAVI) instrument to discriminate the high hydrostatic pressure (HHP) treated Iberian dry-cured *tenderloin* samples from control ones according to various spectral treatments.

Treatment	Pre-treatment	Range (nm)	LVs	n	Calibration		Cross-validation	
					R^2	RMSE	1-VR	RMSECV
HHP	Abs	908,1-1676,2	11	185	0.72	0.265	0.64	0.300
HHP	MSC	908,1-1676,3	15	188	0.78	0.232	0.64	0.300
HHP	SNV + DE	908,1-1676,4	15	169	0.87	0.177	0.76	0.245
**HHP**	**SNV + DE + SG 1,4,4,1**	**908,1-1676,5**	**16**	**161**	**0.87**	**0.182**	**0.78**	**0.233**
HHP	SNV + DE + SG 2,5,5,2	908,1-1676,6	16	172	0.86	0.189	0.76	0.249

HHP = high hydrostatic pressure; Abs = absorbance; MSC = multiplicative scatter correction; SNV = Standard normal variate; DE = de-trending; SG = Savitzky-Golay derivative, with the first number corresponding to order derivative, second and third one indicating the smoothing pints on the left and right sides and the last number corresponding to the polynomial; LVs = latent variables; n = number of samples; 1-VR = coefficient of determination in cross-validation; RMSECV = root mean square error of calibration.

Table S2. PLS-DA calibration results of models developed by means of MicroNIR™ 1700 OnSite-W (VIAVI) instrument to discriminate the Iberian dry-cured *tenderloin* samples preserved at refrigeration temperature from those preserved at room temperature over the course of 8 months.

Treatment	Pre-treatment	Range (nm)	LVs	n	Calibration		Cross-validation	
					R^2	RMSE	1-VR	RMSECV
4ºC	Abs	908.1-1676.2	9	155	0.94	0.123	0.93	0.138
4ºC	MSC	908.1-1676.3	7	155	0.95	0.119	0.93	0.131
4ºC	SNV + DE	908.1-1676.4	7	149	0.95	0.113	0.94	0.124
**4ºC**	**SNV + DE + SG 1,4,4,1**	**908.1-1676.5**	**7**	**149**	**0.95**	**0.110**	**0.94**	**0.122**
4ºC	SNV + DE + SG 2,5,5,2	908.1-1676.6	6	159	0.94	0.118	0.93	0.129

Abs = absorbance; MSC = multiplicative scatter correction; SNV = Standard normal variate; DE = de-trending; SG = Savitzky-Golay derivative, with the first number corresponding to order derivative, second and third one indicating the smoothing pints on the left and right sides and the last number corresponding to the polynomial; LVs = latent variables; n = number of samples; 1-VR = coefficient of determination in cross-validation; RMSECV = root mean square error of calibration.
